# Characterization of non-olfactory GPCRs in human sperm with a focus on GPR18

**DOI:** 10.1038/srep32255

**Published:** 2016-08-30

**Authors:** Caroline Flegel, Felix Vogel, Adrian Hofreuter, Sebastian Wojcik, Clara Schoeder, Katarzyna Kieć-Kononowicz, Norbert H. Brockmeyer, Christa E. Müller, Christian Becker, Janine Altmüller, Hanns Hatt, Günter Gisselmann

**Affiliations:** 1 Ruhr-University Bochum, Department of Cell Physiology, Bochum, Germany; 2 Department of Dermatology, Venerology, and Allergology, University Hospital Essen, University of Duisburg-Essen, Germany; 3 Pharma-Zentrum Bonn, Pharmazeutisches Institut, Pharmazeutische Chemie, Universität Bonn, Bonn, Germany; 4 Jagiellonian University Medical College, Faculty of Pharmacy, Department of Technology and Biotechnology of Drugs, Medyczna 9, PL-30-688 Kraków, Poland; 5 Competence Network for HIV/AIDS, Ruhr University Bochum, Bochum, Germany; 6 Department of Dermatology, Venerology, and Allergology, Center for sexuell health, Ruhr University Bochum, Bochum, Germany; 7 University of Köln, Cologne Center for Genomics, Köln, Germany

## Abstract

G protein-coupled receptors (GPCRs) transduce external chemical cues into intracellular signals and are involved in a plethora of physiological processes, but knowledge regarding the function of these receptors in spermatozoa is limited. In the present study, we performed RNA-Seq and analyzed the expression of the all GPCRs except olfactory receptors in human spermatozoa. We revealed the expression of up to 223 different GPCR transcripts in human spermatozoa (FPKM > 0.1) and identified GPR18, a newly described cannabinoid receptor, together with GPR137 and GPR135, as one of the three most highly expressed GPCRs. To date, the expression of GPR18 was completely unknown in human spermatozoa. We confirmed GPR18 expression using RT-PCR and immuncytochemistry experiments and localized the GPR18 protein in the midpiece of human spermatozoa. Stimulation of human spermatozoa with the GPR18 ligand N-arachidonoylglycine induced the phosphorylation of 12 protein kinases, some of them are for example known to be involved in the acrosome reaction. In line with this, N-arachidonoylglycine affected the cytoskeleton by changing levels of F-actin and inducing the acrosome reaction in human spermatozoa in a concentration-dependent manner. Our results indicate that GPR18 might be involved in physiological processes of human spermatozoa, suggesting GPR18 to be a potential player in sperm physiology.

Millions of spermatozoa are released into the vagina during intercourse. However, only a small number of sperm reach the unfertilized oocyte. On their way to the oocyte, the navigation of mammalian spermatozoa depends on physical and chemical cues^1^. G protein-coupled receptors (GPCRs) comprise the largest family of receptors with seven transmembrane domains and regulate a variety of physiological processes. These receptors detect molecules, such as neurotransmitters, chemokines, hormones or odorants. Known sperm-associated GPCRs are the olfactory receptors. Their functional characterization revealed a physiological role in human spermatozoa^2^,^3^. Nevertheless, until today the investigations of non-olfactory GPCRs in human spermatozoa are rare, and a comprehensive expression analysis of GPCR transcripts from human spermatozoa is lacking.

Cannabinoid receptors belong to the rhodopsin-like family of GPCRs. Two subtypes are known designated CB_1_ and CB_2_. In the last decade, novel GPCRs that can interact with cannabinoids have been identified. One of these receptors is the G protein-coupled receptor 18 (GPR18), which consists of 331 amino acids and is conserved among mammals^4^. GPR18 was initially described at the RNA level in human spleen and testis^4^. Furthermore, GPR18 mRNA was shown to be localized in murine spleen, bone marrow, thymus, lung and cerebellar tissue^5^.

In 2006, *N*-arachidonoylglycine (NAGly) was determined to be an agonist of GPR18^6^. The endogenous lipid NAGly is produced in two different synthesis pathways from the endocannabinoid arachidonoylethanolamine (AEA)^7^,^8^,^9^. Although NAGly has a strong structural resemblance to AEA and 2-arachidonoylglycerol (2-AG), NAGly is not an agonist of the classical cannabinoid receptors CB_1_ and CB_2_^10^. In addition to NAGly, the plant-derived phytocannabinoid Δ^9^-tetrahydrocannabinol (THC) and the structurally related synthetic compounds abnormal cannabidiol (Abn-CBD), O-1602, and O-1918 were described as agonists for GPR18^11^,^12^. Although O-1918 was previously described as an antagonist for GPR18^11^, a recent investigation suggested that O-1918 had an activating effect on GPR18^12^. Recently, the first selective GPR18-antagonists could be identified^13^. Even though several studies suggested that GPR18 was activated by NAGly, some others did not observe an activation of GPR18-expressing cells^13^,^14^. Recently, it was found that GPR18 can show biased activation depending on the employed agonist; for example β-arrestin signaling was only observed upon activation with THC, but not with other agonists^12^.

The female and male genital tract fluids contain significant concentrations of endocannabinoids and other lipids, which suggests that these substances may influence important physiological processes of spermatozoa, such as the regulation of flagellar motion, the switch of the flagellar beating mode, the direction of movement, the capacitation, and the acrosome reaction of spermatozoa^15^,^16^. How these environmental cues are detected and the signals are translated in human sperm has been explored only rudimentarily. Interestingly, NAGly was detected in the female reproductive tract of rat across the hormonal (estrous) cycle^17^.

Various studies with GPR18-expressing cells confirmed the effect of NAGly as an agonist of GPR18^11^,^12^,^18^. So far, GPR18 has been reported to be involved in cellular processes, such as cell migration^11^, apoptosis^19^, regulation of MAPK activity^6^,^11^,^19^,^20^, and immune stimulation^20^. The signaling pathways that are induced upon GPR18 activation are not known in detail. Coupling to Gα_i/o_ was postulated because NAGly-induced effects could be inhibited by pertussis toxin^6^,^11^. Moreover, NAGly induced the phosphorylation of protein kinases, such as p44/42 MAPK^21^.

In this study, we profiled the expression of non-olfactory GPCR transcripts in human spermatozoa by RNA-Seq and identified GPR18 as one of the most highly expressed non-olfactory GPCR transcripts. We investigated the role of GPR18 in human spermatozoa and revealed that the putative endogenous GPR18 agonist alters physiological processes in human spermatozoa.

## Results

### Expression of non-olfactory GPCR transcripts in human spermatozoa determined by RNA-Seq

**Figure 1:**
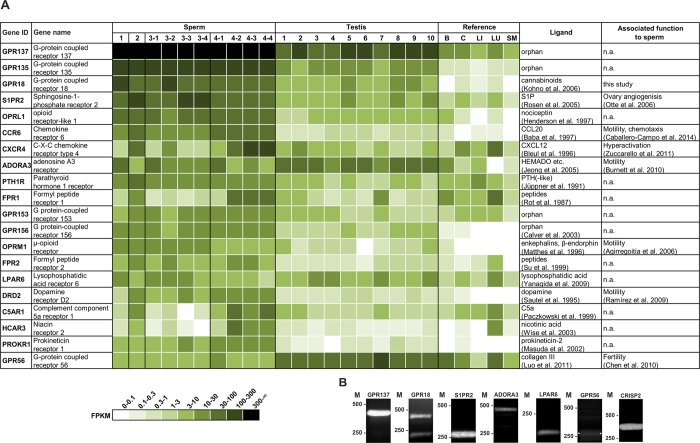
Determination of GPCR transcript levels in human spermatozoa. (**A**) Shown are the 20 most highly expressed GPCR transcripts in human spermatozoa from four different donors. For sperm donors 3 and 4, four different datasets of four independent semen samples each were generated (Sperm 1, 2, 3-1, 3-2, 3-3, 3-4, 4-1, 4-2, 4-3, 4-4) Data were compared to ten different testis samples (Testis 1–10), brain (**B**), colon (**C**), liver (LI), lung (LU) and skeletal muscle (SM). GPCRs are sorted by the mean FPKM in spermatozoa. The FPKM values are an indicator for expression strength of transcripts and are represented by color intensity. Ligand: describes which ligand is known for the respective GPCR. Associated function to sperm: describes whether a function of the respective GPCR is known in sperm. n.a.: not available. (**B**) RT-PCR analysis of GPCRs obtained from RNA-Seq. Gel electrophoresis of the amplified PCR products from cDNA samples of human sperm and testis samples. For GPR18 we detected both transcript variants. The highly expressed sperm-specific transcript CRISP2 was used as a positive control^59^. Used primer pairs were intron-spanning excluding gDNA amplified products. M: Marker in bp.

To investigate the general expression of non-olfactory GPCR transcripts in human spermatozoal RNA samples, we performed next generation sequencing experiments (RNA-Seq). We generated mRNA-sequencing data for 10 human spermatozoa samples from four individual donors (Sperm 1 to 4). For sperm donors 3 and 4, four different datasets of four independent semen samples each were generated. A more detailed description of the transcriptomes will be given elsewhere^22^. The transcriptome analysis revealed the expression of up to 223 out of the 375 different non-olfactory GPCR transcripts in human spermatozoa. A list of all detected non-olfactory GPCR transcripts can be found in Figure S1 (mean FPKM value of all spermatozoa samples = mFPKM > 0.1). We detected for 14 different non-olfactory GPCR transcripts having mFPKM values higher than 30 (GPR137, GPR135, GPR18, S1PR2, OPRL1, CCR6, CXCR4, ADORA3, PTH1R, FPR1, C5AR1, HCAR3, HCAR2, GPR183), and 5 of these transcripts showed expression values higher than 100 mFPKM (GPR137, GPR135, GPR18, S1PR2, CXCR4) in one or more spermatozoa samples. Out of these 223 detected non-olfactory GPCRs, we generated a ranking of the 20 most highly expressed non-olfactory GPCR transcripts in human spermatozoa and compared their expression intensities to testis and different reference tissue samples (brain, colon, liver, lung, skeletal muscle) (Fig. 1A). The three highest mFPKM values were found for GPR137 (1004 mFPKM), GPR135 (208 mFPKM), and GPR18 (86 mFPKM).

Based on FPKM values, GPCR-transcripts were in the same expression range, such as the housekeeping genes GAPDH (842 mFPKM) and RPL29 (219 mFPKM) (Figure S3). GPR137 and GPR135 are orphan receptors. GPR137 is ubiquitously expressed^5^ and has a possible role in tumor cell growth^23^. The GPR135 gene is a target for hypermethylation in ovarian cancer genesis^24^. However, the physiological function of both receptors is unknown. For GPR18, activation by cannabinoids and related lipids has been described^6^,^11^,^12^,^18^. We detected a coherent and high expression for GPR18 transcripts in all of the spermatozoa and testis samples investigated (sperm: a mFPKM of 86.4 (ranging from 38 to 173, testis: a mFPKM 9.7 (ranging from 3 to 25)), whereas the expression of GPR18 transcripts in the reference tissues was low (≤0.52 FPKM), indicating a selective expression in sperm and testis (Figs 1A and S4, S5).

For 7 out of the 20 most highly expressed GPCR transcripts, the function is associated with reproduction or fertilization (Fig. 1A). Most of these already described functions for GPCRs are related to the motility of sperm^25^,^26^,^27^,^28^,^29^.

Furthermore, we performed RT-PCR experiments to confirm the RNA-Seq data of spermatozoa for GPR18 (Figs 1B and S4) as well as for 5 additional GPCRs. We selected GPCR genes with and without a known expression in human sperm from the 20 most highly expressed GPCRs. We confirmed all of the GPCR transcripts detected by RNA-Seq using RT-PCR (Fig. 1B). Furthermore, we confirmed the known transcript variants for GPR18 (Accession number: NM_005292 and NM_001098200) via RT-PCR, concluding that both variants are expressed in human spermatozoa (Fig. 1B).

### GPR18 protein is localized to the midpiece of human spermatozoa

**Figure 2:**
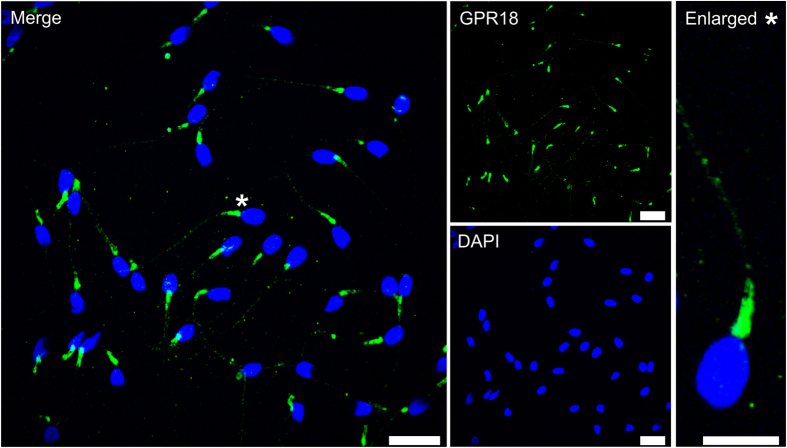
GPR18 protein is localized to the midpiece of human spermatozoa. Immunocytochemical staining of human spermatozoa with an α-GPR18 antibody (green). DAPI staining (blue) was used to determine the number and location of spermatozoa. Scale bars: 10 μm. Enlarged: *indicated the zoomed section. Scale bar: 5 μm.

In this study, we focused on the functional characterization of GPR18 in human spermatozoa because this receptor is the most highly expressed GPCR in human spermatozoa, and the receptor has a known ligand profile. First, we investigated whether GPR18 can be detected at the protein level in human spermatozoa. For the detection of GPR18 proteins, we performed immunocytochemical staining with human spermatozoa. The antibody specificity was previously verified^11^, and the secondary antibody without primary antibody did not produce any specific staining (Figure S6). Apart from mRNA-expression of GPR18 in human spermatozoa via RNA-Seq and RT-PCR, GPR18 was detected at the protein level in all human spermatozoa investigated (Fig. 2). Staining was restricted from the central to distal parts of the midpiece, whereas the head was not stained. In the flagella, we detected weak GPR18 staining.

### The GPR18 ligand NAGly induced the phosphorylation of protein kinases

**Figure 3:**
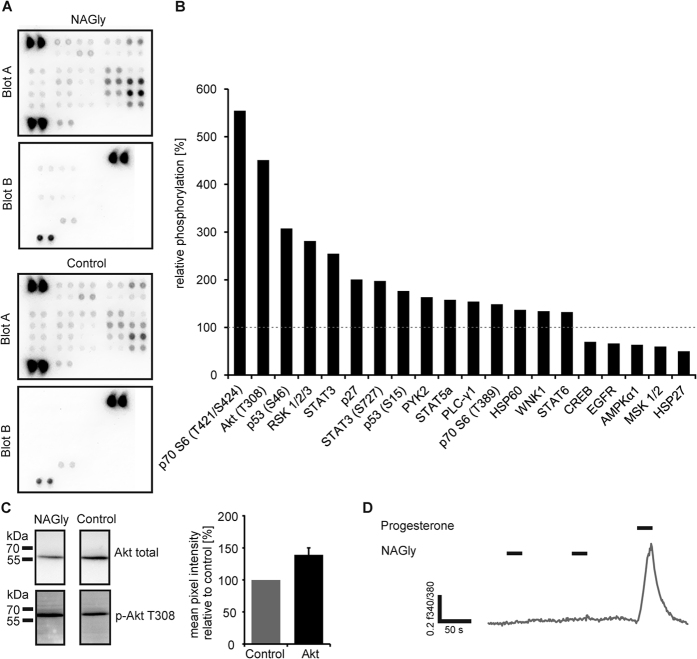
The GPR18 ligand NAGly induced the phosphorylation of different protein kinases in human sperm. (**A**) Human phospho-kinase array analysis revealed phosphorylation events induced by NAGly (3 μM) after 7 min stimulation. (**B**) Shown are the relative phosphorylation (mean pixel intensities relative to control) of two independently performed experiments using two different donor samples. Each array was performed using pooled protein lysates of at least three different semen samples from the same donor. The dotted line indicates the control level. (**C**) Western Blot experiments validated the phosphorylation of Akt at the T308 phosphorylation site. Determination of the total amount of Akt served as a control. Furthermore, the quantification of the mean pixel intensity of phosphorylated Akt relative to total Akt upon NAGly stimulation (black bar) is shown. The ratio was normalized to that observed in DMSO-treated cells (0.1%; control; gray bar). Error bar represents the mean ± SEM (p = 0.016, Student’s t-test, n = 4 experiments). (**D**) In single-cell calcium imaging experiments, the repetitive short-term stimulation with 10 μM NAGly did not induce any calcium signals in human spermatozoa. Cytosolic Ca^2+^ levels are monitored as the integrated f340/f380 fluorescence ratio expressed as a function of time. Black bars indicate the stimulus duration. The positive control (500 nM progesterone) induced strong calcium signals in virtually all sperms and showed the vitality of the sperm and the functionality of the imaging system. In none of the 453 analyzed cells from 4 different sperm samples NAGly induced a Ca^2+^ signal.

To investigate the potential physiological function of GPR18 in human spermatozoa, we examined the effect of the reported GPR18 agonist NAGly in human spermatozoa. Different protein kinases are important regulators of cellular mechanisms. In sperm, the activation of protein kinases is involved in central physiological processes, such as capacitation or acrosomal exocytosis^30^. We investigated the phosphorylation status of 40 different protein kinases upon NAGly stimulation in human spermatozoa using a phospho-kinase array (Fig. 3A). The stimulation of cells with 3 μM NAGly (7 min) induced the phosphorylation of 12 different kinases (Akt, p53, p70 S6, RSK1/2/3, STAT3, p27, PYK2, STAT5a, PLC-γ1, HSP60, WNK1, STAT6). For five kinases, we detected a decreased phosphorylation (CREB, EGFR, AMPKα1, MSK1/2, and HSP27) in comparison to control cells (Fig. 3B). The highest phosphorylation level was observed for the T308 phosphorylation site of the Akt kinase (451%) and the p70 S6 kinase at T421 and S424 (555%) compared with the control.

We used western blot analysis to validate the effect of NAGly on the phosphorylation site T308 of the Akt kinase. We confirmed that NAGly increased Akt phosphorylation after 7 min of agonist exposure. The quantification of western blot experiments validated this significant effect (p = 0.016, Fig. 3C).

An important mediator of processes in spermatozoa, such as the control of hyperactivation, is the induction of changes in calcium concentration (reviewed in ref. 31). Therefore, we performed single-cell calcium imaging experiments with human spermatozoa to investigate if NAGly induces a change of the intracellular calcium concentration in human sperm. Upon short-term application (20 s) of 10 μM NAGly, no increase in intracellular calcium concentrations were observed in human spermatozoa (Fig. 3D). The usage of the control stimulus progesterone (500 nM) induced strong calcium signals.

### NAGly induced a reorganization of the cytoskeleton and enhanced the acrosomal exocytosis in human spermatozoa

**Figure 4:**
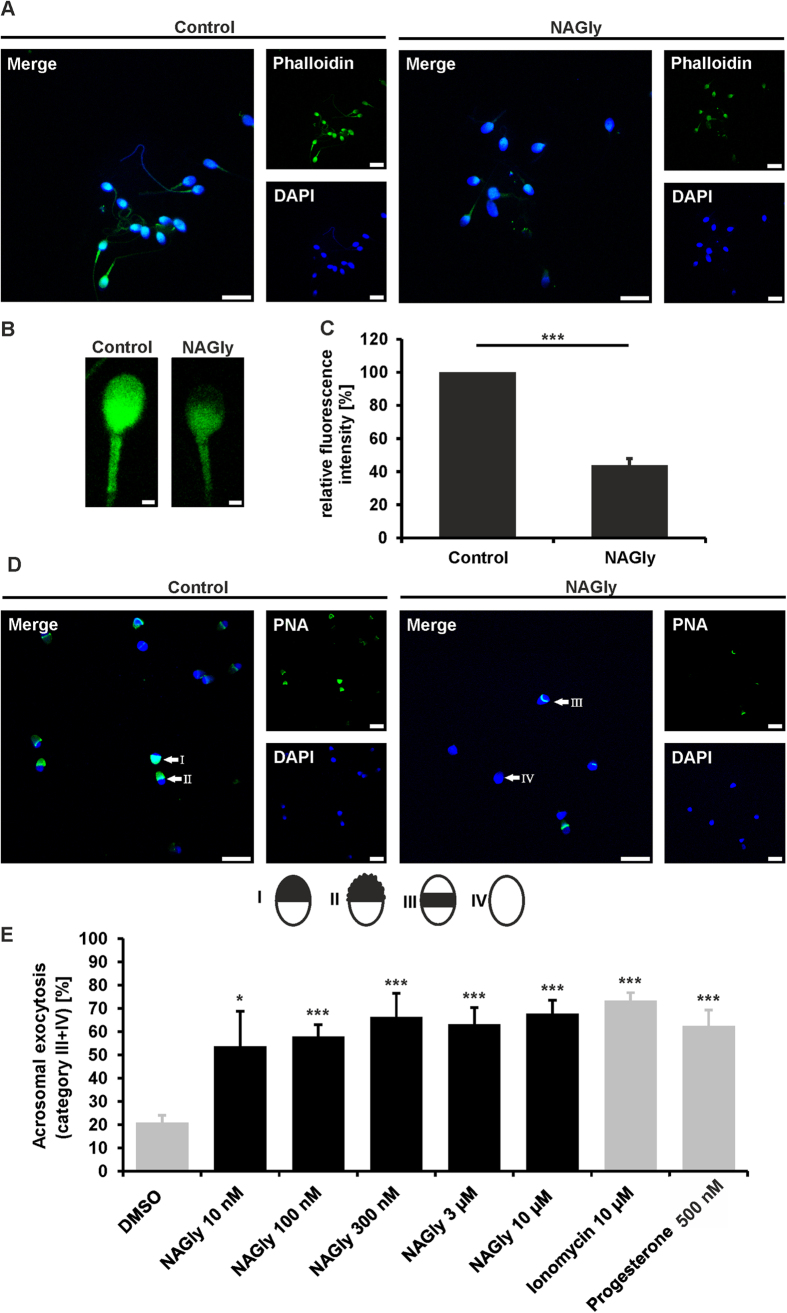
NAGly induced the reorganization of actin filaments and the acrosome reaction in human spermatozoa. (**A**) F-actin staining using FITC-conjugated phalloidin (green) of NAGly-stimulated (3 μM) spermatozoa and control cells (0.1% DMSO). The cells were incubated for 3 h. DAPI staining (blue) was used to determine the number and localization of cell nuclei. Scale bars: 10 μm. (**B**) Zoomed section of NAGly-stimulated (3 μM) and control spermatozoa stained with phalloidin-FITC (Phalloidin). Scale bars: 1 μm. (**C**) Quantification of relative fluorescence intensities revealed a significant reduction of F-actin levels upon NAGly stimulation in human sperm. Shown are the mean ± SEM (DMSO: n = 95 cells; NAGly: n = 95 cells; from three independent experiments; Mann-Whitney-U-Test: ***p < 0.001). (**D**) Representative pictures of the PNA-FITC (PNA) staining of control and NAGly-stimulated cells. Marked are the different categories of the acrosomal status (I-IV). DAPI staining (blue) was used to determine the number and localization of cell nuclei. Scale bars: 10 μm. (**E**) Quantification of acrosomal exocytosis (categories III-IV) upon NAGly stimulation using PNA-FITC. Stimulated cells (NAGly) were compared to control cells (0.1% DMSO); Three to five independent samples with a sperm population ranging from 97 to 364 cells per single experimental condition were analyzed. Values show the mean ± SEM (*p < 0.05, ***p < 0.001, Student’s t-test).

The protein kinases Akt and p70 S6 are involved in actin cytoskeleton dynamicsin human spermatozoa^32^. Furthermore, an involvement of PLCγ in the reorganization of the cytoskeleton was shown^33^,^34^. We investigated the effect of NAGly on cytoskeleton dynamics by detecting F-actin (Fig. 4A). Phalloidin detected F-actin was enriched in the acrosomal region of control cells, defining the profile of the acrosome vesicle. In NAGly-stimulated cells, this staining was significantly reduced, especially in the acrosomal cap (Fig. 4B). After treatment with NAGly (3 μM) for 3 h, the cells showed significant alterations in the cytoskeleton (Fig. 4C).

A decreased amount of F-actin is an indicator of the acrosome reaction in spermatozoa^35^,^36^,^37^. Therefore, we investigated acrosomal exocytosis in human spermatozoa upon NAGly stimulation (Fig. 4D,E). The acrosomal status was divided into four categories (I-IV) as described in ref. 38. Category I corresponds to a complete acrosomal cap, category II to a nearly complete acrosomal cap, category III to a nearly complete acrosomal exocytosis and category IV to complete acrosomal exocytosis; Fig. 4D. After 1 h of NAGly stimulation at different concentrations, we detected a significant concentration-dependent increase of spermatozoa with a nearly complete or a complete acrosomal exocytosis (category III and IV). NAGly (10 μM) was as potent as the positive controls progesterone and ionomycin.

### NAGly- and THC-induced acrosomal exocytosis is mediated by GPR18 in human spermatozoa

**Figure 5:**
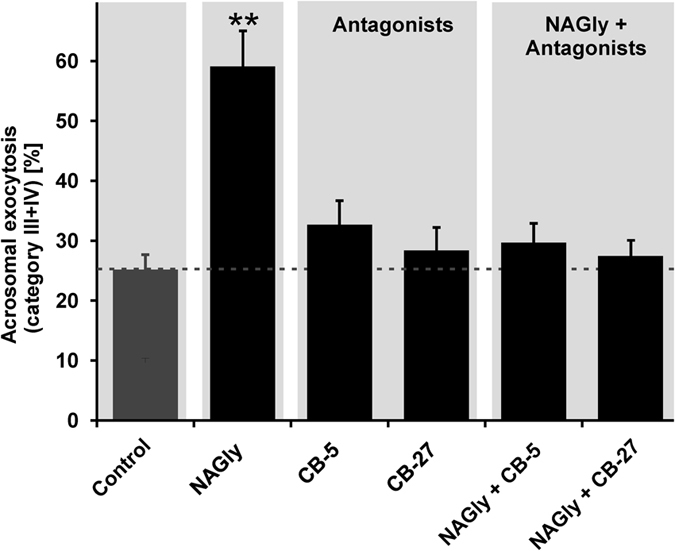
NAGly-induced effects are mediated via GPR18 in human spermatozoa. Quantification of acrosomal exocytosis (categories III-IV) upon co-stimulation of 1 μM NAGly and the selective GPR18 antagonists PSB-CB5 (1 μM) and PSB-CB27 (1 μM) using PNA-FITC. Stimulated cells (NAGly + antagonist) were compared to control cells (0.1% DMSO); four independent samples with a sperm population ranging from 143 to 260 cells per single experimental condition were analyzed. Values show the mean ± SEM (**p < 0.01, Student’s t-test).

To confirm that NAGly-induced effects are mediated by GPR18 activation, we used specific GPR18 antagonists^13^ (Supporting Information). The co-application of the selective GPR18 antagonist PSB-CB5 and PSB-CB27 with NAGly (1:1, 1 μM) completely blocked the NAGly-induced acrosomal exocytosis in human spermatozoa (Fig. 5). The results showed that the NAGly-induced acrosomal exocytosis is mediated by the activation of GPR18 in spermatozoa. Furthermore, we investigated the effects of the phytocannabinoid THC (Figure S7). The cannabinoid also induced the acrosomal exocytosis. Again, these effects could be inhibited by the specific GPR18 antagonists PSB-CB5 and PSB-CB27.

## Discussion

GPCRs form the largest family of transmembrane signaling receptors and regulate a plethora of physiological and pathophysiological processes. To our knowledge, this is the first comprehensive expression analysis of non-olfactory GPCR transcripts in motile human spermatozoa. We revealed the expression of 223 different non-olfactory GPCRs. For 7 out of the 20 most highly expressed GPCRs, the expression as well as the function was already described in sperm, indicating the significance of these RNA-Seq data sets. In addition, we described the expression of newly identified non-olfactory GPCR-transcripts in spermatozoal RNA. Thirteen out of the twenty most highly expressed GPCRs were not described in human spermatozoa so far.

We detected a high level of GPR18 expression for the first time in human spermatozoa. GPR18 is the most highly expressed GPCR for which ligands are known. Additionally, we demonstrated GPR18 expression at the protein level in the midpiece of spermatozoa using immunocytochemical staining.

Many effects of GPR18 in different types of cells were previously shown^11^,^18^,^19^. However, the functional role of GPR18 in spermatozoa has so far not been determined.

The agonist of GPR18, NAGly, induced the activation of several protein kinases in spermatozoa. This activation might be involved in actin bundling in human spermatozoa, as well as in the induction of the acrosome reaction. Stimulation with the GPR18 ligand induced a strong phosphorylation of Akt and p70 S6 kinases. Furthermore, PLCγ was phosphorylated upon NAGly stimulation. These protein kinases play regulatory roles in actin polymerization in human sperm^32^,^34^,^39^.

During the physiological reorganization of sperm that happens between ejaculation and fertilization, the structure of actin in the sperm cells changes several times. After ejaculation, G-actin monomers are predominantly present in sperm. After capacitation in the female genital tract, this G-actin is polymerized to F-actin. This process increases the motility of the cells^36^,^40^,^41^,^42^,^43^. As part of the last stages of acrosomal exocytosis, which is triggered upon reaching the ovum, the F-actin is cleaved again to G-actin (Liu *et al*.^35^, 2002, Brener *et al*.^36^). This is a necessary process that enables the outer acrosomal membrane and the plasma membrane to come into close proximity and fuse^44^. NAGly stimulation led to the depolymerization of F-actin and induced the acrosomal exocytosis in human sperm. This indicates that the NAGly activation of GPR18 is important for the physiology of sperm just it penetrates the oocyte.

Preliminary data indicated no chemotactic effect upon NAGly stimulation using a capillary chemotaxis assay as described in ref. 45. An effective influence on the motility was not be proven. The effect of NAGly stimulation on the flagellar beat frequency should be investigated in future experiments.

NAGly is an endogenous lipid that is found throughout the human body, including the female reproductive tract. In the uterus of rodents, varying concentrations of NAGly were detected during the hormonal cycle (estrus: ~40 pmol/gram uterus tissue)^17^. The highest concentration of NAGly was cycle-dependently detected before and during ovulation^46^. In addition, it was demonstrated that the expression of fatty acid amide hydrolase, which catalyzes the synthesis of endogenous NAGly^9^, also varies in the female menstrual cycle of rats^47^. Furthermore, the GPR18 agonist NAGly induced migration of endometrial cells^21^. Thus, future studies should identify if changes in NAGly levels in the endocannabinoid system in human females might influence the effectiveness of a successful fertilization. However, appropriate studies on the presence of NAGly in the female genital tract of humans are still lacking.

The use of the specific GPR18 antagonists showed the direct GPR18 involvement in the agonist-induced effects (NAGly, THC) observed in this study. We can therefore exclude the involvement of cannabinoid CB_1_ or CB_2_ receptors in the detected NAGly- and THC-induced effects in human spermatozoa.

However, we cannot exclude that the NAGly-induced effects in human spermatozoa may be partially mediated by lipid receptors distinct from GPR18, CB_1_ or CB_2_. But the expression of other known or putative cannabinoid-like and lipid receptor transcripts is extremely low (Figure S8). Nevertheless, a second described NAGly receptor LPAR5 (GPR92), is also expressed in human spermatozoa (mFPKM = 1.5), but at a much lower level than GPR18. Additionally, LPAR5 can only be partially activated by higher concentrations of NAGly (EC_50_ = 4.5 μM)^48^,^49^, whereas GPR18 can be activated by lower concentrations in stably transfected HEK293 cells (EC_50_ = 44.5 nM)^21^. In the present study, we also showed that low NAGly concentrations (10 nM) induced acrosomal exocytosis, suggesting that LPAR5 is not involved in NAGly-induced effects. Known antagonists for LPAR5^50^ could be tested in future experiments to completely exclude the potential involvement of LPAR5 in NAGly-induced effects in sperm.

In addition to the endogenous lipid NAGly, we and others could show that GPR18 can be activated by the phytocannabinoid THC^12^,^13^,^21^,^51^,^52^,^53^. A previous study showed that THC attenuates mouse sperm motility^54^. Further studies should investigate if the use of marijuana influences reproduction via GPR18 in males as well as in females. It would be conceivable that activation of GPR18 would have an effect in the male reproductive tract (early sperm acrosome reaction or sperm motility) as well as in the female reproductive tract (activation of GPR18 independently of the regular hormonal cycle).

## Conclusions

RNA-Seq analysis of human spermatozoa revealed the expression of 223 different non-olfactory GPCR transcripts. One of the most highly expressed GPCRs was the NAGly-sensitive GPR18. Stimulation with NAGly and thus the activation of GPR18 is involved in key physiological processes of human spermatozoa. NAGly induced the reorganization of actin filaments and induced acrosomal exocytosis. Together, the GPR18 activation might play an important role, particularly immediately prior to fertilization. Our findings indicate the involvement of a new cannabinoid receptor in male reproduction.

## Materials and Methods

### Human semen collection preparation

Human sperm were freshly obtained from young healthy donors who gave informed signed consent. Samples were used anonymously. Sperm collection and analysis was performed according to the local regulations and approved by the local ethics committee of the Ruhr-University Bochum (Reg.-Nr. 2231). For RNA isolation, Ca^2+^ imaging, acrosome assay, F-actin staining, western blot experiments, Phospho-Kinase array, and immuncytochemistry experiments, motile spermatozoa were obtained as follows. After liquefaction (30 min at 35.5 °C) to isolate mature and motile sperm, a Percoll density gradient centrifugation was performed as described previously^2^. Liquefied semen was overlaid on a two-layer Percoll (cell culture tested, Sigma-Aldrich, MO, USA) density gradient and centrifuged at room temperature for 40 min at 300 g. The pellet was collected, washed in standard Ringer’s solution (140 mM NaCl, 5 mM KCl, 2 mM CaCl_2_, 2 mM MgCl_2_, 10 mM Hepes, 10 mM glucose, pH 7.4), and again centrifuged for 15 min. Then, the pellet of motile spermatozoa was resuspended in Ringer’s solution and used for further experiments.

### RNA isolation

The isolation of spermatozoal RNA was performed using the RNeasy Plus Mini Kit (Qiagen, Hilden, Germany) according to the manufacturer’s protocol including DNaseI digestion.

### Transcriptome analysis

Basic NGS analysis was done as previously described^22^. Briefly, for transcriptome analysis RNA from human motile spermatozoa was prepared as stated above. At the Cologne Center for Genomics Next Generation Sequencing unit, libraries for next generation sequencing were constructed from mRNA. RNA-Seq was performed on the Illumina HiSeq2000 sequencing platform as paired-end reads with 101-nucleotide length. Raw sequence data were aligned to the human reference genome hg19 using the TopHat software. BAM-files were sorted and indexed using the Samtools software package^55^. FPKM (fragments per kilobase of exon per million fragments mapped) values were calculated using the Cufflinks software. For the current study, we extracted the values for GPCRs out of this data set. A comprehensive description of the transcriptome of spermatozoa was given elsewhere^22^. For comparison to spermatozoa transcript expression, we reanalyzed already published raw data in the same manner as RNA-Seq data of spermatozoa samples. We reanalyzed data from 8 different testis samples obtained from the Array Express Archive (www.ebi.ac.uk/arrayexpress/; accession number: E-MTAB-1733). The five reference tissues as well as Testis 1 were obtained from the Body Map 2.0 project from the NCBI GEO database (http://www.ncbi.nlm.nih.gov/gds/; accession number: GSE30611). Raw data of Testis 2 were obtained from^56^.

### Reverse transcriptase (RT)-PCR

RNA from motile spermatozoa was reverse transcribed using the iScript cDNA Synthesis Kit (Bio-Rad Laboratories, Hercules, CA, USA) according to the manufacturer’s instructions. An equivalent of ~30 ng of RNA was used for each RT-PCR experiment. We designed exon-exon spanning primers that detect ~150–450 bp of the respective gene (Figure S2). PCR was performed using GoTaq qPCR Master Mix (Promega, Madison, WI, USA) with the Mastercycler realplex^2^ (Eppendorf, Hamburg, Germany) (20 μl total volume, 40 cycles: 95 °C, 59 °C, 72 °C, 45 s each). All experiments were conducted in triplicate.

### Phospho-Kinase Array

The Proteome Profiler Human Phospho-Kinase Array (ARY003; R&D Systems, Inc., MN, USA) was used to detect kinase phosphorylation according to the manufacturer’s protocol. In brief, motile sperm were stimulated with 3 μM NAGly or the equivalent amount of DMSO (0.1%) for 7 min. Afterwards, proteins were isolated and 200 μg protein was applied to each array set. In this method, proteins are captured by antibodies spotted on a nitrocellulose membrane. Levels of phosphorylated protein are then assessed using an HRP-conjugated antibody followed by chemiluminescence detection. The amount of chemiluminescence was detected and analyzed with the ImageJ software using a microarray plug in ref. 57.

### Western Blot Analysis

Sperm cells were treated with 3 μM NAGly for 7 min, pelleted by centrifugation at 500 g for 5 min, washed with PBS, and homogenized in lysis buffer (50 mM Tris/HCl, pH 7.4, 150 mM NaCl, 1 mM EDTA, and 1% Triton X-100) with protease and phosphatase inhibitors (PhosSTOP Phosphatase Inhibitor Cocktail Tablets, Roche, Basel, Switzerland). Western Blot analyses were performed as previously described^58^. The following primary antibodies were used: Akt total, phospho-Akt T308 (Cell Signaling Technology). Antibodies were diluted (1:1000) in 3% blocking reagent in Tris buffered saline + 0.1% Triton-X-100. After washing, membrane was incubated with HRP-coupled secondary antibody and followed up with chemiluminescence detection (ECL Select Western Blotting Detection System, Amersham Biosciences, GE Healthcare, Solingen, Germany). The data were quantified using ImageJ software^57^. The ratio of the average intensity of the phosphorylated sample band and the average intensity of unphosphorylated sample band exhibited the relative intensity.

### Immunocytochemical staining

For immunocytochemical staining, motile spermatozoa plated on poly-l-lysine coated cover slips were fixed with 4% paraformaldehyde (20 min, room temperature). Afterwards, cells were permeabilized with PBS^−/−^ + 0.1% Triton X-100 and incubated with blocking reagent (PBS^−/−^ + 0.1% Triton X-100, 5% normal goat serum, and 1% fish gelatin) for 1 h. Primary antibody was incubated overnight (4 °C). The following antibody was used: α-GPR18, 1:100 (kindly provided from Ken Mackie, Indiana University). Antibody-specificity was proven in ref. 11. Alexa-conjugated secondary antibody (Alexa Fluor 488 Goat Anti-Rabbit, Invitrogen, Denmark) was incubated for 45 min at room temperature in the absence of light. Probes were mounted in ProLong Gold antifade (Invitrogen, Denmark). Images were obtained using a confocal fluorescent microscope (LSM 510 Meta, Zeiss).

### Calcium imaging experiments

Calcium imaging experiments with human spermatozoa were performed as described previously in ref. 2.

### Acrosome assay

For analysis of acrosome reactions sperm were incubated with the respective substance for 1 h at 37 °C. As control, sperm were incubated in 0.1% DMSO. Next, 100 μl of the sperm suspension was transferred on a microscope slide. The samples were air-dried and fixed for 20 min in 4% paraformaldehyde at room temperature. For acrosome staining, samples were incubated with 5 μg/ml peanut agglutinin (PNA)-FITC (Sigma-Aldrich, MO, USA) in PBS^−/−^ for 45 min in the dark at room temperature. During this incubation, sperm were counterstained with DAPI (Invitrogen, Denmark). Samples were mounted in ProLong Gold antifade (Invitrogen, Denmark). Slides were analyzed using a confocal fluorescent microscope (LSM 510 Meta, Zeiss).

### F-actin staining

For the analysis of cytoskeletal changes, sperm were incubated with NAGly (3 μM) for 3 h at 37 °C. As a control, sperm cells incubated with an equivalent of DMSO (0.1%), were used. After stimulation, cell suspensions were transferred to poly-l-lysine-coated cover slips in 24-well plates. After washing with Ringer’s solution, cells were fixed using 4% PFA for 20 min at room temperature. Cells were incubated for 5 min with PBST (PBS^−/−^ + 0.1% Triton X-100), after two washes with PBS^−/−^. For F-actin staining, cells were incubated with Phalloidin-FITC (50 μg/ml, Invitrogen, Denmark) in PBST for 20 min at room temperature in the dark. During this incubation, a counterstaining with DAPI was performed. After washes with PBS^−/^, samples were mounted in ProLong Gold antifade (Invitrogen, Denmark).

### Test compounds

Appropriate stock concentrations of the chemicals tested were prepared in 100% DMSO. The desired final working concentrations were achieved by serially diluting the stock solutions with Ringer’s solution (each containing 0.1% DMSO).

### Statistical analysis

Statistical analysis was performed using SigmaPlot 12.3. The results are presented as the mean ± standard error of mean (SEM) and (n) is the number of cells/experiments. The significance was set as *p < 0.05, **p < 0.01, ***p < 0.0001.

## Additional Information

**How to cite this article**: Flegel, C. *et al*. Characterization of non-olfactory GPCRs in human sperm with a focus on GPR18. *Sci. Rep*. **6**, 32255; doi: 10.1038/srep32255 (2016).

## Supplementary Material

Supplementary Information
