# Supplementary material for: Characterization of non-olfactory GPCRs in human sperm with a focus on GPR18
Source: Sci Rep. 2016 Aug 30;6:32255. doi: 10.1038/srep32255 (PMC5004183; doi:10.1038/srep32255)
Supplement: Supplementary Information [file srep32255-s1.pdf]

SUPPORTING INFORMATION

Characterization of non-olfactory GPCRs in human sperm with a focus on GPR18

Caroline Flegel[1], Felix Vogel[1,5], Adrian Hofreuter[1], Sebastian Wojcik[1], Clara Schoeder[3], Katarzyna Kieć-Kononowicz[4], Norbert H. Brockmeyer[6,7], Christa E. Müller[3], Christian Becker[2], Janine Altmüller[2], Hanns Hatt[1], Günter Gisselmann[1]

[1]Ruhr-University Bochum, Department of Cell Physiology, Bochum, Germany
[2]University of Köln, Cologne Center for Genomics, Köln, Germany
[3]Pharma-Zentrum Bonn, Pharmazeutisches Institut, Pharmazeutische Chemie, Universität Bonn, Bonn, Germany
[4]Jagiellonian University Medical College, Faculty of Pharmacy, Department of Technology and Biotechnology of Drugs, Medyczna 9, PL-30-688 Kraków, Poland
[5]Department of Dermatology, Venerology, and Allergology, University Hospital Essen, University of Duisburg-Essen, Germany
[6]Competence Network for HIV/AIDS, Ruhr University Bochum, Bochum, Germany
[7]Department of Dermatology, Venerology, and Allergology, Center for sexuell health, Ruhr University Bochum, Bochum, Germany

SUPPORTING INFORMATION

GPCR	Class	mFPKM
GPR137	Other	992.8
GPR135	Class A	204.6
GPR18	Class A	91.7
S1PR2	Class A	78.7
OPRL1	Class A	38.3
CCR6	Class A	31.2
CXCR4	Class A	29.5
ADORA3	Class A	25.6
PTH1R	Class B	21.0
FPR1	Class A	17.8
GPR153	Class A	17.8
GPR156	Class C	13.4
OPRM1	Class A	11.2
FPR2	Class A	10.3
LPAR6	Class A	9.9
DRD2	Class A	9.8
C5AR1	Class A	9.2
HCAR3	Class A	9.1
PROKR1	Class A	8.6
GPR56	Adhesion	7.6
HCAR2	Class A	7.5
OPN3	Class A	7.3
GRM8	Class C	6.5
OPN5	Class A	6.4
MC1R	Class A	6.3
P2RY2	Class A	5.8
LPAR3	Class A	4.9
CHRM4	Class A	4.8
TAS1R1	Class C	4.8
FFAR2	Class A	4.8
C3AR1	Class A	4.5
MRGPRF	Class A	4.4
GPR97	Adhesion	4.4
CCRL2	Class A	4.3
LPHN3	Adhesion	4.3
PTAFR	Class A	4.0
MTNR1A	Class A	4.0
GPR183	Class A	3.8
GPR84	Class A	3.8
GRM5	Class C	3.4
GPR160	Class A	3.4
GPR85	Class A	3.3
GPR65	Class A	3.1
CCR1	Class A	3.1
GPR182	Class A	3.0
HTR7	Class A	3.0
ELTD1	Adhesion	2.9
ADORA2A	Class A	2.8
CELSR2	Adhesion	2.7
GPRC5B	Class C	2.4
CXCR2	Class A	2.4
SSTR2	Class A	2.2
FZD9	Frizzled	2.2
CHRM5	Class A	2.1
LGR6	Class A	2.1
GPR176	Class A	2.1
CXCR1	Class A	2.0
CD97	Adhesion	2.0
HTR5A	Class A	1.9
GPR107	Other	1.9
HCAR1	Class A	1.8
CHRM2	Class A	1.7
DRD3	Class A	1.7
GRM7	Class C	1.6
GRM3	Class C	1.6
FSHR	Class A	1.5
EMR2	Adhesion	1.5
GPR45	Class A	1.5
LPAR5	Class A	1.5
PTGER4	Class A	1.4
VIPR1	Class B	1.4
BAI2	Adhesion	1.3
TAS1R3	Class C	1.3
GPR125	Adhesion	1.3
NMBR	Class A	1.3
BAI3	Adhesion	1.2
GABBR2	Class C	1.2
EMR1	Adhesion	1.2
XCR1	Class A	1.1
MCHR2	Class A	1.1
GPR87	Class A	1.0
FPR3	Class A	1.0
NPY5R	Class A	1.0
TPRA1	Other	1.0
GPR133	Adhesion	1.0
GPR34	Class A	1.0
PTGER3	Class A	1.0
GPR158	Class C	0.9
P2RY14	Class A	0.9
GPRC5D	Class C	0.9
FZD6	Frizzled	0.9
FZD4	Frizzled	0.8
GPR19	Class A	0.8
GNRHR2	Class A	0.8
HTR1D	Class A	0.8
CHRM3	Class A	0.8
CCR5	Class A	0.7
GPR68	Class A	0.7
EMR3	Adhesion	0.7
LPHN1	Adhesion	0.7
F2RL1	Class A	0.7
LGR5	Class A	0.7
GPR98	Adhesion	0.7
P2RY13	Class A	0.7
BAI1	Adhesion	0.7
GABBR1	Class C	0.6
P2RY6	Class A	0.6
HTR4	Class A	0.6
FZD3	Frizzled	0.6
LPAR2	Class A	0.6
LTB4R	Class A	0.6
HRH2	Class A	0.6
CCR7	Class A	0.6
GPR114	Adhesion	0.6
HTR2A	Class A	0.6
GPR25	Class A	0.6
GPR63	Class A	0.6
ADRA1A	Class A	0.6
CALCR	Class B	0.6
DRD4	Class A	0.5
CHRM1	Class A	0.5
CELSR3	Adhesion	0.5
CELSR1	Adhesion	0.5
GPBAR1	Class A	0.5
P2RY12	Class A	0.5
LGR4	Class A	0.5
PTGER2	Class A	0.5
LPAR1	Class A	0.5
GPR35	Class A	0.5
F2R	Class A	0.5
GPRC5A	Class C	0.5
GPR37L1	Class A	0.5
EDNRB	Class A	0.4
GPR113	Adhesion	0.4
EMR4P	Adhesion	0.4
GPRC5C	Class C	0.4
TSHR	Class A	0.4
CYSLTR2	Class A	0.4
EDNRA	Class A	0.4
GPR123	Adhesion	0.4
GALR3	Class A	0.4
GPR4	Class A	0.4
FZD7	Frizzled	0.4
SUCNR1	Class A	0.4
NPFFR1	Class A	0.4
ADRB2	Class A	0.4
GRM2	Class C	0.4
PTH2R	Class B	0.4
UTS2R	Class A	0.3
GRM1	Class C	0.3
CMKLR1	Class A	0.3
MC5R	Class A	0.3
MC2R	Class A	0.3
ADRA2A	Class A	0.3
GPR132	Class A	0.3
GPR110	Adhesion	0.3
LTB4R2	Class A	0.3
GPR161	Class A	0.3
CASR	Class C	0.3
NPY1R	Class A	0.3
CCR10	Class A	0.2
S1PR5	Class A	0.2
CXCR6	Class A	0.2
CCKAR	Class A	0.2
NMUR1	Class A	0.2
CYSLTR1	Class A	0.2
NPY6R	Class A	0.2
FFAR3	Class A	0.2
PTGFR	Class A	0.2
DARC	Class A	0.2
P2RY11	Class A	0.2
CRHR1	Class B	0.2
GRM4	Class C	0.2
CCR3	Class A	0.2
CRHR2	Class B	0.2
GPR75	Class A	0.2
P2RY10	Class A	0.2
GPR39	Class A	0.2
MRGPRX3	Class A	0.2
GPR143	Other	0.2
GPR141	Class A	0.2
GPR12	Class A	0.2
NPFFR2	Class A	0.2
TAAR2	Class A	0.2
CX3CR1	Class A	0.2
GPR151	Class A	0.2
CCR9	Class A	0.2
GPR64	Adhesion	0.2
S1PR4	Class A	0.2
HTR1B	Class A	0.2
BDKRB1	Class A	0.2
GPR22	Class A	0.2
OXGR1	Class A	0.2
HRH1	Class A	0.2
CCR2	Class A	0.2
GPR116	Adhesion	0.2
OXTR	Class A	0.2
MRGPRD	Class A	0.1
GPR115	Adhesion	0.1
P2RY8	Class A	0.1
GPR20	Class A	0.1
GPR146	Class A	0.1
MAS1	Class A	0.1
ADORA2B	Class A	0.1
BRS3	Class A	0.1
CCR4	Class A	0.1
FZD5	Frizzled	0.1
SSTR4	Class A	0.1
P2RY1	Class A	0.1
GPR61	Class A	0.1
NPSR1	Class A	0.1
GRM6	Class C	0.1
GPR162	Class A	0.1
CCKBR	Class A	0.1
GPR171	Class A	0.1
FZD1	Frizzled	0.1
GPR157	Other	0.1
GPR83	Class A	0.1
AVPR1A	Class A	0.1
GPR62	Class A	0.1
NPBWR1	Class A	0.1
GPR33	Class A	0.1
GPR3	Class A	0.1

Figure S1. All detected non-olfactory GPCR transcripts in human sperm (mFPKM >0.1).

Gene		Primer Sequence (5' - 3')	Product length [bp]
**ADORA3**	fwd	TCCTTCCAGTCATGTGGCTC	452
**ADORA3**	rev	CTTGCGGACAACTTTGGGAG	
**CRISP2**	fwd	AGTTGCAAGTGCAAAGGGAG	360
**CRISP2**	rev	CAATTCCACAGCCTACCTGG	
**GPR137**	fwd	CGTCTCAGCTATCAGACGGT	394
**GPR137**	rev	AGATGACGAACAGGGAGTCG	
**GPR18**	fwd	GCCAAGCGTTACACTGGAAA	217/
**GPR18**	rev	CAGCTTGTTGGTAGGCATGA	409
**GPR56**	fwd	TCCTGAAGCATCCCCAGAAG	299
**GPR56**	rev	GAGTCTCTTCTCAGCCTCCC	
**LPAR6**	fwd	TCTGCTATGGCTCTTCCTCAG	152
**LPAR6**	rev	GAGGCCTTTTCCTCAGTTGC	
**S1PR2**	fwd	CCTGGGGACGCAGACG	259
**S1PR3**	rev	AGGTTTTCCACCACAATGGC	

Figure S2. Primer sequences used for RT-PCR validation qPCR.

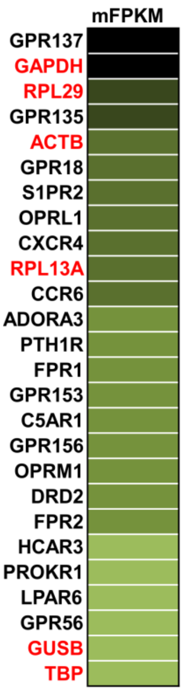

Figure S3. Expression of GPCR transcripts in comparison to housekeeping gene transcripts in human spermatozoa. Shown are the mFPKM values for the 20 most highly expressed GPCR in comparison to several housekeeping genes in spermatozoa. Transcripts are sorted by the mFPKM. Housekeeping genes are marked in red. RPL29: ribosomal protein L29, ACTB: ß-actin; RPL13A: ribosomal protein L13A; GUSB: β-glucuronidase; TBP: TATA box binding protein.

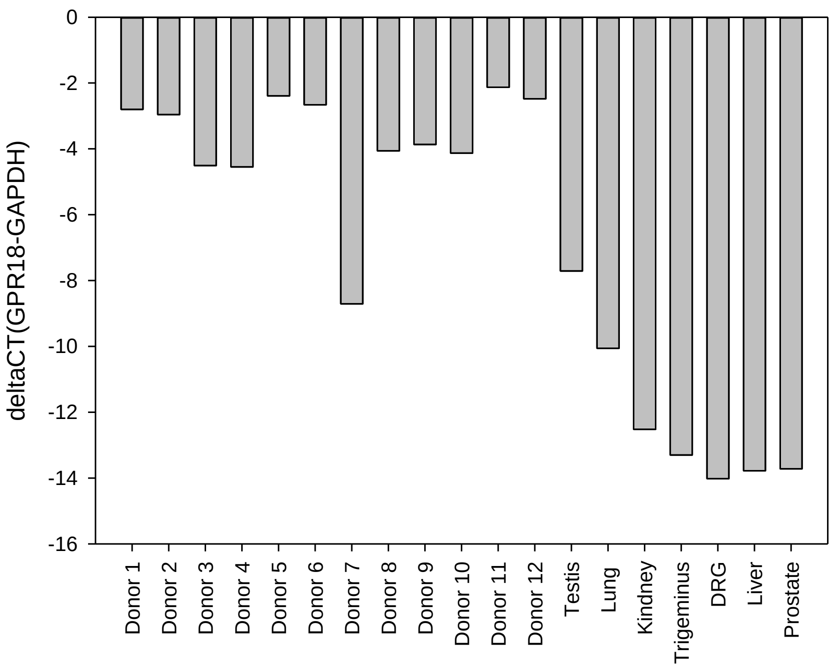

Figure S4. Detection of GPR18 transcripts by RT-PCR. The sperm RNA of different donors and RNA from different tissues was investigated by real time RT-PCR basically as described [1] using intron-spanning primer pairs for GPR18 (Figure S2) and GAPDH (fw: ACCACAGTCCATGGCCATCAC, rv: TCCCACCACCCTGTTGCTGTA). The deltaCT values for the sperm samples (typically between -3 and - 5) point to a considerably higher relative transcript abundance in sperm compared to the control tissues.

[1] Flegel et al., PLoS One. 2015 Jun 12;10(6):e0128951

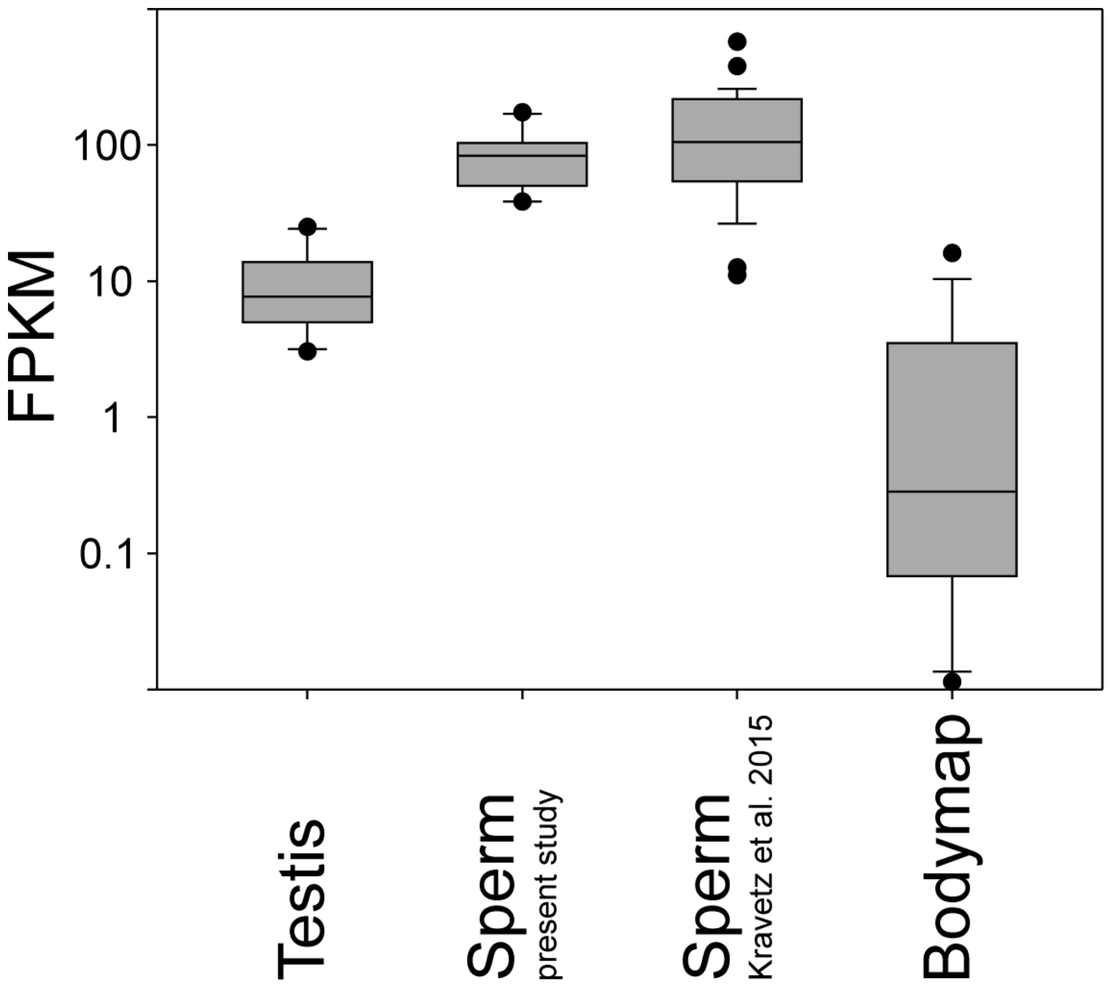

Figure S5. Detection of GPR18 transcripts by NGS. Recently, a comprehensive NGS-based study of RNA expression in sperm was conducted analyzing 72 samples (GEO dataset GSE65683) [1]. We reanalyzed the raw fastq data sets of 28 of these samples with the same parameters as used for our own NGS data sets and found a similar distribution of the FPKM values similar to our own generated data. GPR18 was expressed in these samples with a mean FPKM of 139 (min = 11, max = 573), not statistically different from the FPKM values calculated for our own sperm samples (mean FPKM of 86, min = 38, max = 173) ($p > 0.05$, Student's t-test). However, in both sperm data sets the FPKM values are significantly higher ($p>0.0001$) than in testis and in the 16 reference tissues of the Bodymap Project [2]. With the exception of one sample (white blood cells), in this reference data set all FPKM values are lower than the lowest FPKM found for GPR18 in sperm.

[1] Kravetz et al., Sci Transl Med. 2015 Jul 8;7(295):295re6

[2] Flegel et al., PLoS One. 2013;8(2):e55368

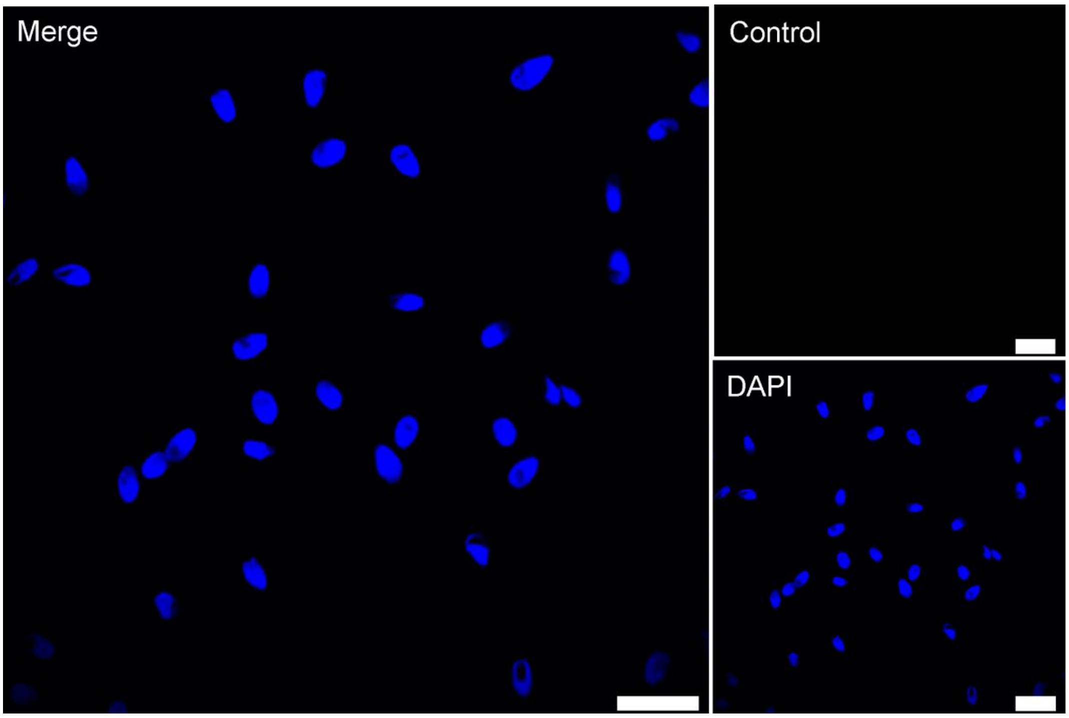

Figure S6. Secondary antibody control in human spermatozoa. As a negative control, staining was performed without primary antibody, showing no specific staining by the secondary antibody (Alexa Fluor 488 Goat Anti-Rabbit, (Control). Scale bar: 10 µm.

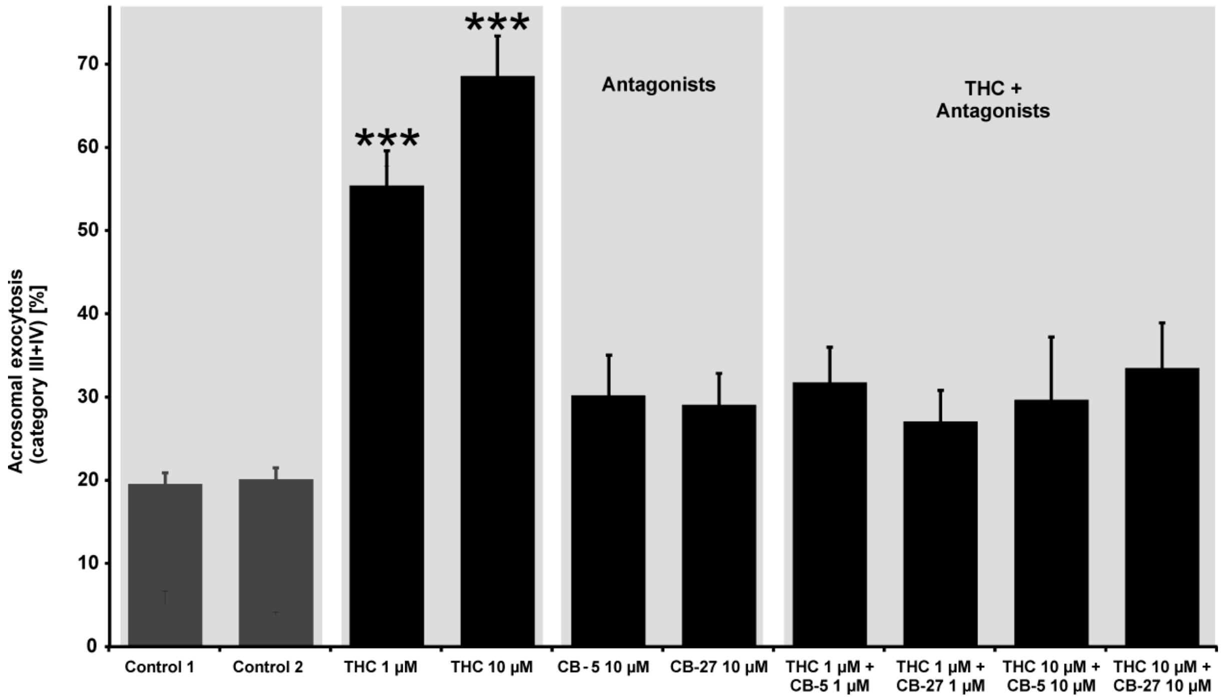

Figure S7. Cannabinoid-induced effects are mediated by GPR18 in human spermatozoa. Quantification of acrosomal exocytosis (categories III-IV) upon co-stimulation of THC and the selective GPR18-antagonists PSB-CB5 and PSB-CB27 (1:1, 1 µM or 10 µM) using PNA-FITC. Initial experiments showed that also the phytocannabinoid THC induced the acrosome reaction in human spermatozoa via GPR18. Stimulated cells (cannabinoid + antagonist) were compared to control cells (0.1 % DMSO). Three to five independent samples were analyzed with a sperm population ranging from 87 to 264 cells per single experimental condition. Values show the mean ± SEM (***$p < 0.001$, Student's t-test).

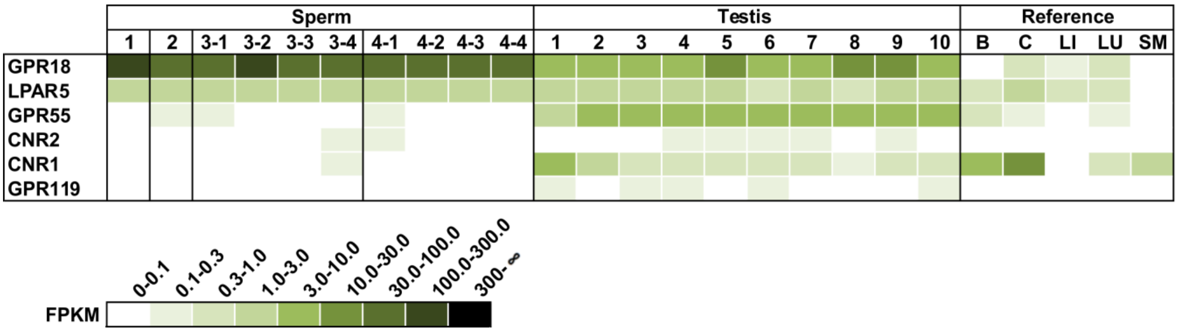

Figure S8. Cannabinoid receptor transcripts in human spermatozoa. Heatmap of known cannabinoid receptor transcripts.

Supporting Data

GPR18 antagonists

PSB-CB5, (*Z*)-2-(3-(4-Chlorobenzyloxy)benzylidene)-6,7-dihydro-2*H*-imidazo[2,1-*b*][1,3]-thiazin-3(5*H*)-one[1]

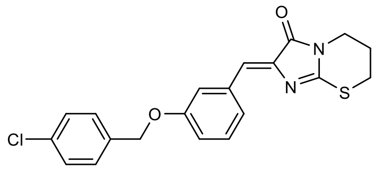

PSB-CB27, (*Z*)-2-(3-(6-(4-Chlorophenoxy)hexyloxy)benzylidene)-6,7-dihydro-2*H*-imidazo-[2,1-*b*][1,3]thiazin-3(5*H*)-one

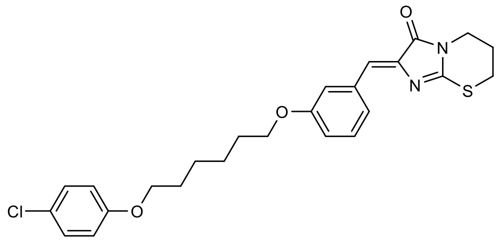

Table 1. Potencies of GPR18 inhibitors PSB-CB5 and PSB-CB27 for GPR18, GPR55, CB_1 and CB_2 receptors. All data result from three independent experiments, performed in duplicate. (For experimental details see below)[1]

compound	Inhibition of agonist-induced β-arrestin recruitment		Radioligand binding vs. $[^3H]$CP55,940	
	**human GPR18**	**human GPR55**	**human CB_1**	**human CB_2**
	IC_{50} ± SEM (µM)		K_i ± SEM (µM)	
PSB-CB5[1]	**0.279** ± 0.111	>10 (0%)	>10 (0%)	4.03 ± 0.51
PSB-CB27	**0.650** ± 0.134	>10 (37%)	>10 (-14%)	>10 (12%)

While PSB-CB5 showed incomplete inhibition of THC-induced GPR18 activation (58% maximal inhibition), PSB-CB27 led to a complete inhibition of GPR18-induced β-arrestin translocation.

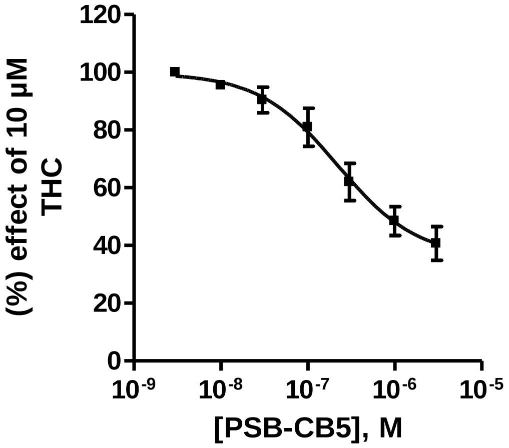

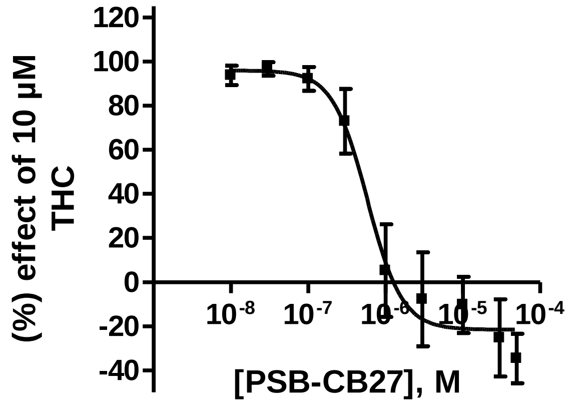

Figure 1: Concentration-dependent inhibition of THC- (10 µM) -induced GPR18 activation by the GPR18 antagonists PSB-CB5 and PSB-CB27.

Method

β-Arrestin recruitment assay

Recruitment of β-arrestin to the respective receptor was detected by using β-galactosidase enzyme fragment complementation technology (β-arrestin PathHunter™ assay, DiscoverX, Fremont, CA, USA). CHO cells stably expressing the respective receptor were seeded in a volume of 90 µL into a 96-well plate and were incubated at a density of 20,000 cells/well in assay medium (Opti-MEM, 2 % FCS, 100 U/ml penicillin, 100 µg/ml Streptomycin, 800 µg/mL geneticin und 300 µg/mL hygromycin) for 24 h at 37°C. Then test compounds were diluted in PBS buffer containing 10 % DMSO and 0.1 % BSA and added to the cells in a volume of 10 µL, followed by an incubation for 90 min at 37° C. For determination of baseline luminescence PBS buffer (containing 10 % DMSO, 0.1 % BSA) in the absence of test compound was used. During the incubation period, the detection reagent was prepared. For determination of β-arrestin recruitment to GPR18 the provided detection reagent was used, according to the supplier's protocol. The composition of the detection reagent for GPR55 assays was changed: it was obtained by mixing the chemiluminescent substrate Galacton-StarR (2 mM), with the luminescence enhancer Emerald-II™ and a lysis buffer (10 mM TRIS, 1 mM EDTA, 100 mM NaCl, 5 mM $MgCl_2$, 1 % Triton-X; pH 8) in a ratio of 1:5:19. After the addition of 50 µl/well of detection reagent to the cells, the plate was incubated for further 60 min at room temperature. Finally luminescence was determined in a luminometer (TopCount NXT, Packard / Perkin-Elmer).

Reference for supporting data

[1] Rempel, V.; Atzler, K.; Behrenswerth, A.; Karcz, T.; Schoeder, C.; Hinz, S.; Kaleta, M.; Thimm, D.; Kiec-Kononowicz, K.; Müller, C. E. Med. Chem. Comm. 2014, 5, 632.
